# Comparison of Methods to Assess Adherence to Infant and Young Child Feeding Practices and Provision of Low-Aflatoxin Porridge Flours in a Community-Based Intervention Trial

**DOI:** 10.3390/nu16244315

**Published:** 2024-12-13

**Authors:** Erica Phillips, Rosemary A. Kayanda, Neema Kassim, Francis M. Ngure, Paul C. Turner, Rebecca J. Stoltzfus

**Affiliations:** 1 Department of Nutritional Sciences, University of Wisconsin-Madison, Madison, WI 53706, USA; 2 Division of Nutritional Sciences, Cornell University, Ithaca, NY 14850, USA; fmn9@cornell.edu; 3 Department of Food Biotechnology and Nutritional Sciences, Nelson Mandela African Institution of Science and Technology, Arusha 23311, Tanzania; 4 Global, Environmental, and Occupational Health, University of Maryland, College Park, MD 20742, USA; pturner3@umd.edu; 5 Office of the President, Goshen College, Goshen, IN 46526, USA

**Keywords:** adherence, infant and young child feeding, community-based intervention, community survey, 24 h dietary recall

## Abstract

Background: Levels of adherence to recommended protocols in an intervention trial can affect outcomes and confound the results. To broaden the evidence about the selection and utility of adherence measures in varying contexts, we describe the level of adherence to the Mycotoxin Mitigation Trial (MMT) randomized intervention using caregiver-reported survey questions and compare inferences of adherence between multi-module surveys and interactive 24 h dietary recalls based on our program theory. Methods: The MMT was a two-arm cluster-randomized trial conducted in 52 health facilities (clusters) in central Tanzania. Surveys were conducted with all trial participants at three time points and dietary recalls were conducted in a cohort at 12 mo. Results: The 12 mo survey was conducted with 2112 caregivers and the 18 mo survey was conducted with 2527 caregivers. A cohort of participants (*n* = 282, 20 clusters) was selected for dietary recalls, balanced by arm. Reported feeding of blended porridge flours, whether MMT-provided or own-sourced, was high at 12 and 18 mo, between 73 and 95%, with only slight differences between the surveys and recalls. Inferences were similar for continuation of breastfeeding, feeding frequency, and dietary diversity. Only the amount of porridge fed the previous day differed statistically by method, with higher amounts reported in the recalls compared to the survey. Conclusions: Detailed analysis of reported behaviors, based on the MMT program theory, supports high adherence to the recommended trial behaviors. Survey data and 24 h dietary recalls were convergent for almost all indicators, strengthening the trial’s conclusions and allowing for either method to be selected for similar research.

## 1. Introduction

The application of theory-driven approaches in the development, monitoring, and evaluation of nutrition interventions and research is increasingly used to derive a better understanding of why, how, and under what conditions objectives are or are not achieved [1,2,3,4,5]. Theory-driven approaches can generate data used for analysis and nuanced interpretation of research findings, providing a high level of confidence in the results and refinement of future research development and design [6,7].

Adherence to recommended intervention behaviors and the facilitators and barriers to these are core components of program theory [8,9,10]. However, the reporting of adherence measures varies widely, despite recommended reporting guidelines and being part of the CONSORT guidelines [11,12,13,14]. Adherence is a determinant of intervention outcomes; low adherence can reduce an intervention’s effectiveness, confounding results in research and minimizing potential health benefits in practice [15,16]. While there is a robust body of literature about adherence in medication interventions, there has been less focus on the definition, assessment, analysis, and reporting of adherence within infant and young child feeding (IYCF) research and programs.

ICYF interventions are often multi-component and increasingly multi-sectoral [17]. They may include a behavior communication change (BCC) component and/or provide a new resource, such as complementary or supplementary foods, lipid-based supplements (LNSs), or micronutrients in various forms. The hypothesized impact pathways of these programs differs from pharmaceutical interventions, requiring adaptation in how adherence is defined and measured.

Unlike other assessments in health and nutrition, there is no “gold standard” for measuring adherence [9,18]. Methods to estimate adherence are often descriptively referred to as “subjective” or “indirect” and “objective” or “direct” [18,19]. Subjective or indirect measures are often self-reported through surveys, diaries, journals, or dietary recalls. Objective or direct measures can include observation, measurement or tracking of prescribed items in various ways, and clinical measures using biomarkers or metabolites.

All adherence measures have advantages and disadvantages, and their ultimate selection depends on their reliability, validity, and potential biases, in addition to practical considerations such as available resources and capabilities. For example, maternal self-report of diet and infant feeding behaviors is an indirect method frequently used for IYCF interventions, as it does not require high economic resources. In some studies, self-report has been found to modestly overestimate actual behavior, whereas in other studies using a longer reporting period, self-reporting underestimated actual behavior [20,21,22,23].

Common direct measures include the calculation of disappearance rates or observation, which can be more reliable but also expensive, intrusive, and time-consuming. For some nutrition outcomes, a validated biomarker might be available as a direct measure of adherence, but these can be affected by diet, absorption, and rate of excretion, and can be expensive. Given these advantages and disadvantages, and the varying contexts in which adherence might be measured, the following general principles for assessing adherence are often recommended: (a) stating an operational definition of adherence a priori, (b) the use of indicators that are specific to the behaviors being studied, and (c) triangulation of subjective and objective measures [8].

The MMT was a community-based two-arm cluster-randomized trial conducted in central Tanzania between 2019 and 2021 to assess the effect of aflatoxin (AF) consumption on linear growth between 6 and 18 mos of age [24,25]. The trial was conducted following multiple observational studies showing a positive association between AF consumption and reduced linear and ponderal growth in children under 5 years of age [26,27,28,29]. The internal validity of the MMT depended on creating a contrast of AF consumption between arms through receipt and feeding of low-AF maize and groundnut flours in the intervention arm, while maintaining typical and comparable feeding practices between arms. Defining, measuring, and analyzing adherence to recommended feeding practices was critical to causal inference.

To help broaden the evidence about the assessment and analytic use of adherence measures and indicators of IYCF programs, the aims of this paper are to (a) describe the level of adherence to recommended IYCF behaviors using multi-module survey data within the Mycotoxin Mitigation Trial (MMT), and (b) compare results of the survey data with 24 h dietary recalls conducted in a cohort of trial participants.

## 2. Methods

### 2.1. Trial Participants and Intervention

Infant/mother dyads were enrolled into the MMT over one year from their home health facility (i.e., cluster) between 6 weeks and 3 months of age using Well-Baby attendance records. The intervention included IYCF education sessions offered to mothers enrolled in both trial arms by MMT-trained community health workers when their infants were 4, 5, 8 and 11 months old. These were scheduled to be timely for dietary changes during the first year of life. The randomized intervention began when the enrolled infant turned 6 mos of age. In the intervention arm, a low-AF pre-blended porridge flour (maize and groundnut) and separate low-AF groundnut flour were provided to mothers. In the standard-of-care (SoC) arm, a locally popular skin lotion of similar monetary value was distributed. The same porridge flours were promoted through education but procured by the household in the SoC arm. The low-AF flours (intervention) and skin lotion (SoC) were distributed monthly by research staff at health facilities on a pre-determined day. All mothers were given a vacuum flask to store cooked porridge and a plastic scoop to measure the recommended amount of porridge flour. There was no attempt to mask the treatments in this study as all parties could see what was received by the mother. Due to cluster randomization, all mothers at a given clinic received the same intervention. Detailed description of the formative research, intervention design, and trial outcomes have been published elsewhere [24,30,31,32,33].

### 2.2. Data Collection

All enrolled mothers were surveyed by trained data collectors at recruitment and when the infant was 6, 12, and 18 mo of age. The recruitment survey included modules about parent and household demographics and the infant’s health and care. The 6, 12, and 18 mo surveys were conducted at health facilities and consisted of multiple, identical modules about the infant’s health, care, and feeding practices, maternal diet, and anthropometric assessment. Data were captured using hand-held tablets (Samsung Galaxy 7 (Sourced in Arusha, Tanzania), KoBoToolbox Platform, https://www.kobotoolbox.org/about-us/software/, accessed on 18 November 2024), checked for quality, and uploaded daily.

Between July and December 2020, a sub-cohort of dyads was randomly selected from 20 clusters, chosen for accessibility, to participate in quantitative 24 h dietary recalls [34]. These recalls were conducted in households by two trained enumerators, typically within 4 weeks of the clinic-based survey at 12 mo of age, using an interactive, five-pass method [35]. Dietary recalls were recorded by hand and entered into CSDietary (version 2.0, HarvestPlus and SerPro S.A. 2020, https://www.intake.org/resource/csdietary-software-program, accessed on 19 November 2024) by the two enumerators then exported for statistical analysis. An updated food composition table for Central and East Uganda was used for the majority of recipes and foods, with some adaptation for local recipes [36]. The Tanzanian food composition table or United States Department of Agriculture (USDA) database was used to fill in under 10% of foods not found in the Ugandan table [37,38].

### 2.3. Definition and Operationalization of Adherence

**Figure 1:**
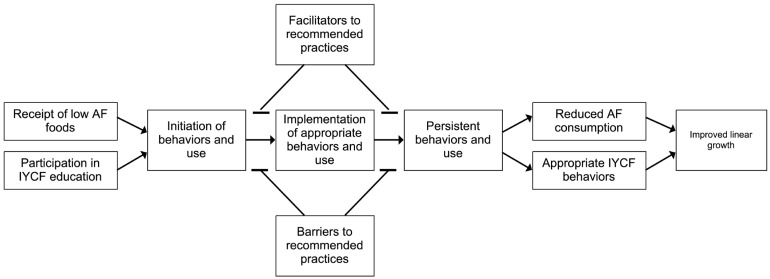
Adherence section of Program Impact Pathway (PIP).

In the formative research phase of the project, we developed a conceptual model of adherence to the intervention as part of a larger Program Impact Pathway (PIP) (Figure 1). Key dimensions, or phases, of adherence, adapted from the medication adherence literature, included (1) initiation, (2) implementation, and (3) persistence, or sustained IYCF behaviors for all mothers and use of the provided MMT flours for those in the intervention arm. To operationalize these dimensions of adherence, outcomes aligned with trial recommendations (Table 1). For the initiation dimension, we assessed whether porridge made from blended cereal and groundnut flour was fed to all infants in the past 24 h and whether MMT-produced porridge flour was fed in the intervention arm in the past 24 h. For the implementation dimension, we measured whether the infant was breastfed at 12 mo of age, the feeding frequency, the amount of porridge fed (mL), and the number of food groups fed for all infants. In the intervention arm, further assessment of implementation included the number of times the caregiver cooked the infant MMT porridge the previous week and behaviors around sharing of MMT flours within and outside of the household. To evaluate persistence of behaviors, we used the two indicators from the 18-mo survey that matched the initiation indicators from the 12 mo survey.

### 2.4. Statistical Analysis

Using all available data from the 12 and 18 mo surveys and 24 h dietary recalls at 12 mo, we described and compared adherence indicators by method and between arms. We accounted for clustering of dyads within health facilities for reported maternal and infant characteristics and adherence measures (STATA svyset command). We used linear and logistic regressions to generate predicted point estimates and probabilities and 95% CIs by arm for continuous and binary outcomes, respectively. Based on our objectives, we report only unadjusted models. All statistical analyses were performed with STATA (version 16.1).

## 3. Results

The 12 mo survey was conducted between March 2020 and February 2021, with a 2 mo stoppage in data collection between April and May 2020 due to the SARS-CoV-2 pandemic. Seventy-four percent (2112/2842) of enrolled mothers (*n* = 1032 intervention arm; *n* = 1080 SOC arm; 26 clusters per arm) participated in the 12 mo survey. The 24 h recall cohort included 282 infants (*n* = 140 intervention arm; *n* = 142 SoC arm; 10 clusters per arm), of which 279 had 12 mo survey data. The 18 mo survey was conducted between September 2020 and August 2021 with 1233 and 1294 participants in the intervention and SoC arm, respectively (26 clusters per arm), which was 88.9% of enrolled mothers and balanced by arm.

The average age of infants in the 12 mo survey was 1 year and 5 days (SD 21.7 days) compared to 1 year and 2 days (SD 20 days) in the recalls (Table 2). There was a slightly higher proportion of females and infants born at a low birthweight (LBW) in the survey dataset compared to the recall dataset, although LBW was notably low in this population. There were no significant differences in mean anthropometric indicators between the survey and recall population at the 12 mo time point. The average maternal age at recruitment was 26.6 years (SD 7.3; range 16–51) for mothers who participated in the 12 mo survey and 27.1 (SD 7.5; range 16–46) for those who participated in 24 h recalls.

### 3.1. Initiation of Recommended Behaviors

In the 12 mo survey, 90% of mothers in the intervention arm and 83% in the SoC arm (*p* < 0.01) reported porridge feeding using a blended cereal and groundnut mixture the previous day (Table 3). According to the dietary recalls, 95% of mothers in the intervention arm and 83% in the SoC arm fed any blended cereal and groundnut flours (*p* < 0.01). In total, 73% of mothers reported feeding MMT-provided flours in the intervention arm in the 12 mo survey, compared to 95% in the 24 h recalls.

### 3.2. Implementation of Recommended Behaviors

The point estimates and whether there was a statistical difference between arms were similar for both methods for continuation of breastfeeding (98–99%) and the number of food groups fed the previous day (Table 3). The reported feeding frequency did not differ statistically between arms for either the survey or recalls, but the point estimates were qualitatively inconsistent between the two methods; the 12 mo survey had an average of 3.2 feeding occasions compared to 4.6 in the recalls. The estimated mean consumption of porridge by infants the previous day according to the survey was 316 mL in the intervention arm and 267 mL in the SoC arm (*p* < 0.01). In the recalls, intervention-arm mothers estimated their infant consumed 338 mL and 313 mL in the SoC arm (*p* = 0.28).

**Figure 2:**
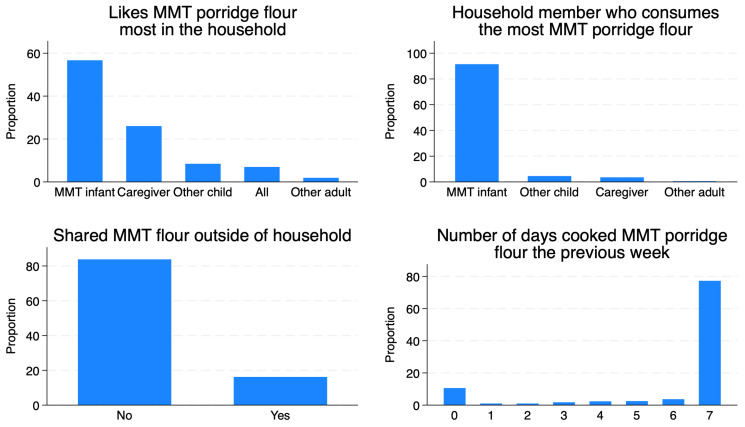
Maternal-reported sharing practices of MMT-provided porridge flours (intervention arm only).

In total, 92% of mothers reported that the index child consumed the most MMT porridge flour in the household, and under 10% of mothers responded that another child or caregiver consumed more porridge than the index child (Figure 2). In the intervention arm, 10.6% of mothers reported not using the provided pre-blended porridge at all during the week prior to the survey, while 76.2% reported using the flour seven times the previous week. For those who did not cook porridge the previous day, the most frequently reported reasons were running out of the provided ration by the time of the survey, traveling outside the household, that the infant was ill, and not having sufficient ingredients to mix with the flours.

### 3.3. Persistence/Sustained Behaviors

According to the survey data at 18 mo, 87% of mothers in the intervention arm fed porridge from a cereal and groundnut blend compared to 76% of mothers in the SoC arm (*p* < 0.01). The percent of intervention arm mothers who fed cereal and groundnut porridges dropped by 3% between the 6 mo and 12 mo surveys, compared to an 8% decrease in the SoC arm. Seventy three percent of mothers in the intervention arm fed their infant with MMT-produced porridge flour according to the 18-mo survey, the same as reported at 12 mos.

## 4. Discussion

The internal validity of randomized intervention trials hinges on participant adherence to recommended behaviors. In the MMT, general IYCF messages were provided equally to mothers in both study arms following formative research to design locally acceptable and feasible recommendations in this setting [30,31,32]. This analysis showed that infants enrolled in the MMT had high levels of initiation, implementation, and persistence, with few differences in feeding behaviors between arms. When comparing survey data to the highly structured 24 h recalls, the majority of dimensions of adherence yielded similar inferences.

For six of the nine indicators used to assess adherence at 12 mo, we were able to compare the results to the 24 h recalls. The three non-comparable indicators were outside of the 24 h reference period of the dietary recalls. The two methodologies resulted in similar statistical inferences for five of the six indicators; only the amount of porridge fed the previous day differed significantly between arms in the surveys but not the recalls. This was likely due to the different physical measures used to estimate consumption. The surveys were conducted at health facilities and enumerators carried a variety of common, local bowls and cups used for porridge feeding. Mothers selected the one closest to what was used in their home to estimate intake. The 24 h recalls were conducted in homes using mothers’ own kitchen measures, potentially making estimates more accurate [35]. Additionally, participant and/or enumerator fatigue from the nine module surveys conducted at the health centers could have resulted in underestimates of porridge consumption. In a multi-module survey in Ethiopia, fatigue was found to reduce women’s dietary diversity scores by 8–17% when the module was placed later in the survey [39]. In rural Ghana, participant fatigue from a multi-module survey reduced reporting of household member activities for those later in the roster [40].

Feeding of MMT-produced flour in the intervention arm and the number of estimated feeding occasions on the previous day resulted in the same statistical inference between methods, but with different point estimates. The surveys and distribution of flours or lotion was conducted once per month at each facility. At the 12-mo survey, the mother would have received the previous ration of flours around 30 days prior. Each ration was estimated to last 30 days, however given variation in use by households, it is not surprising that about a quarter of mothers did not cook with MMT-provided flour the previous day. The dietary recalls, however, were conducted at random times during the month, not all at the end of the month, when all mothers reported having stock of the provided flours at home.

The lower reported feeding occasions in the survey data are likely attributed to the cognitive complexity of asking a mother to estimate the overall number of feeding occasions without prompts or breaking down the question into smaller segments [41]. By contrast, the multi-pass 24 h recall method used multiple prompts and means to break down of consumption by feeding by episode [35]. Similarly, asking mothers to estimate how long it took to finish the previous month’s ration resulted in unreliable responses, with 16% of mothers unable to provide any estimate. These questions could have been more effective if broken down by a shorter reference period and prompts [23].

Surveys and dietary recalls are considered indirect measures of adherence, while biomarkers are direct measures of adherence. There are validated biomarkers of AF exposure, but our operational definition of adherence focused on IYCF practices, with the biomarkers considered partially mediated by adherence [42]. AF is a naturally occurring, odorless, colorless, and tasteless environmental toxin; children in both study arms could have unintentionally and unwittingly ingested AF through family foods, even while adhering to the IYCF recommendations.

A number of studies and programs that distributed LNS and micronutrient powders have compared methods to assess adherence, often combined with studying acceptance in formative research [43,44,45]. In Malawi, comparison between maternal self-report and lab analysis of fortified oil added to porridge found consistent results between methods, although the authors note the bias of mothers reporting the amount that they were taught to use rather than actual usage [20]. In Burkina Faso, both caregiver self-report and disappearance rates based on used packets showed high adherence to LNS, but home observation and plasma zinc concentration suggested low adherence [21]. In Nepal, there was a high correlation between the number of micronutrient sachets reported to have been consumed and the number of unopened sachets shown to the data collector [22].

When interventions provide novel products for infants, a common concern is that they will be shared among household members. Sharing may reduce the amount fed to the target child and may increase the cost of the intervention. However, sharing only interferes with nutritional outcomes if it results in less of the intended product being consumed by the enrolled child. Although reported as “sharing”, leftovers given to household members would not affect the nutritional outcomes of a study, nor would sharing of a “buffer” ration [46,47]. We attempted to minimize meaningful sharing of MMT flours by providing mothers with a product most appropriate for the age of the enrolled infant, not older family members, and by providing a buffer portion of porridge flour of 10–15 g per day (13–20% of the daily ration for a 12–18 mo old).

To assess sharing behaviors, we attempted to design survey questions that minimized courtesy or social desirability bias. Data collectors asked indirectly about sharing by inquiring about others in the household who enjoy the taste of the product and the amount other members consume relative to the enrolled infant. Although many other household members had tasted and enjoyed the products, the enrolled infant was reported to consume most of the provided flours in over 90% of households. The 24 h recalls confirmed (through a multi-step process) that the amount of flour used to make porridge was close to the recommended range of 60 g/day for 9–11 month olds and 75 g/day for 12–18 month old children.

The limitations of this study provide useful learning opportunities for future research design. We did not directly observe household feeding practices, which could have been beneficial. However, direct observations can be demanding of staff and participant time, intrusive, and potentially not reflective of long-term behaviors. To obtain a more reliable disappearance rate, we could have weighed the remaining MMT porridge flours when in households. We chose not to do this (nor ask mothers to bring any remaining flours back to clinics) to avoid sowing mistrust between research staff and participants. Finally, because dietary recalls are time-intensive, we selected a subset of participants, approximately 10% of the recruited sample, who might not represent the full sample. However, we compared all adherence indicators for the subset of mothers to all 12 mo survey respondents and did not observe any differences [34].

## 5. Conclusions

Results from this study support a high level of adherence to the MMT IYCF recommendations and strengthen the internal validity of the trial. The use of two methods to assess adherence allowed for triangulation of results and deeper understanding of adherent and nonadherent behaviors. Comparison of indicators for the three dimensions of adherence between survey results and dietary recalls resulted in similar conclusions. Survey data allowed us to ask questions over a longer reference period and about general household behavior, while 24 h recall data generated granular data about recipes and nutrient intake over a short time horizon.

The integration of the taxonomy and reporting guidelines from the medication adherence literature into theory-driven approaches applied to nutrition research could fill important knowledge gaps about the strengths and weaknesses of various adherence methods as well as context-specific applications. In line with reporting guidelines for research, such as CONSORT and STROBE, more consistent and transparent publication of adherence methods and results would build a more rigorous body of research to facilitate informed decision-making throughout the research planning, implementation, and analysis phases. It would also allow for stronger comparisons between studies and ultimately lead to improved health outcomes.

## Figures and Tables

**Table 1:** Dimensions of adherence, caregiver behaviors, and data sources.

Dimension of Adherence by Arm	Behavior	Data Source		
		12 mo survey	24 h dietary recall	18 mo survey
Initiation				
Feeding behaviors (both arms)	Infant fed porridge made from cereal and groundnut blended flour	X	X	
Use of supplement (intervention arm)	Infant fed with MMT-produced porridge flour in intervention arm	X	X	
Implementation				
Feeding behaviors (both arms)	Infant continues to breastfeed	X	X	
	Infant fed with appropriate frequency	X	X	
	Infant consumed appropriate amount of porridge	X	X	
	Infant consumed foods from multiple food groups	X	X	
Use of supplement (intervention arm)	Caregiver cooked MMT porridge flour 7 days the previous week	X	X	
	Caregiver reported that other household members tasted flours	X	X	
	Caregiver reported sharing flours outside the household	X	X	
Persistence/sustained behaviors				
Feeding behaviors (both arms)	Infant fed porridge made from cereal and groundnut blended flour			X
Use of supplement (intervention arm)	Infant fed with MMT-produced porridge flour in intervention arm			X

**Table 2:** Maternal and infant characteristics.

Maternal-Reported Behavior	12-Month Survey		24 h Dietary Recall	
	Intervention Arm	SoC Arm	Intervention Arm	SoC Arm
Infant Characteristics	1023–1032	1067–1080	140	139
Infant age in days, mean (SD)	369.5 (20.2)	370.5 (23.0)	366.1 (23.1)	366.9 (15.6)
Female sex	522/1032 (50.6%)	550/1080 (50.9%)	65/139 (46.8%)	62/140 (44.3%)
If low birthweight, indicated on child health card	22/792 (2.8%)	25/778 (3.2%)	2/110 (1.8%)	2/103 (1.9%)
Length for age Z score, mean (SD)	−1.5 (1.0)	−1.6 (1.0)	−1.7 (0.9)	−1.5 (1.0)
Weight for age Z score, mean (SD)	−0.8 (1.1)	−0.9 (1.1)	−0.9 (1.1)	−0.8 (1.0)
Weight for length Z score, mean (SD)	−0.0 (1.1)	−0.1 (1.1)	0.0 (1.1)	−0.1 (1.0)
Maternal characteristics (at recruitment visit)				
Maternal age, mean (SD)	26.5 (7.3)	26.7 (7.4)	27.1 (7.4)	27.2 (7.6)
Currently married	786/1032 (76.2%)	811/1080 (75.1%)	112/140 (80.0%)	107/139 (77/0%)
Parity, mean (SD)	3.3 (2.0)	3.4 (2.2)	3.3 (2.0)	3.5 (2.3)
Maternal education				
No schooling/did not complete primary school	461/1032 (44.7%)	498/1080 (46.1%)	61/140 (43.6%)	47/139 (33.8%)
Completed primary school	511/1032 (49.5%)	530/1080 (49.1%)	72/140 (51.4%)	85/139 (61.2%)
Completed secondary school	60/1032 (5.8%)	52/1080 (4.8%)	7/140 (5.0%)	7/139 (5.0%)

Notes: SD—standard deviation, SoC—standard of care.

**Table 3:** Maternal-reported dietary and porridge feeding behaviors by arm and method of data collection, previous day’s consumption.

Maternal-Reported Behavior for Infant	Corresponding Education Message (General IYCF vs. MMT-Specific ^†^)	12-Month Survey			24 h Dietary Recall			18-Month Survey		
		Intervention	Control	*p*	Intervention	Control	*p*	Intervention	Control	*p*
Mother/child dyads		1020–1032	1052–1080		140	139		1233	1294	
Initiation of behaviors										
Fed cereal/groundnut blended flour porridge	MMT #1	89.7% (87.6, 91.8)	82.6% (79.9, 85.4)	<0.01 *	95.0% (91.0, 99.0)	83.0% (75.2, 90.9)	0.01 *	N/A	N/A	N/A
Fed MMT-produced porridge flour—intervention arm	MMT #2	73.3% (70.1, 76.4%)	N/A	N/A	95.0% (91.2, 98.8%)	N/A	N/A	N/A	N/A	N/A
Implementation of behaviors										
Infant continues to breastfeed	IYCF #1	99.1% (98.4, 99.8)	99.2% (98.6, 99.7)	0.93	99.3% (98.0, 100)	97.8% (95.4, 100)	0.31	N/A	N/A	N/A
Number of feeding occasions	IYCF #2	3.3 (3.3, 3.4)	3.2 (3.1, 3.3)	0.16	4.6 (4.2, 4.9)	4.6 (4.3, 5.0)	0.81	N/A	N/A	N/A
Amount of porridge fed previous day (in mL)	MMT #3	316 (299, 333)	267 (251, 284)	<0.01 *	338 (313, 363)	312 (271, 354)	0.28	N/A	N/A	N/A
Infant consumes multiple food groups	IYCF #3	4.9 (4.8, 4.9)	4.8 (4.7, 4.9)	0.51	4.8 (4.6, 4.9)	4.7 (4.6, 4.9)	0.38	N/A	N/A	N/A
Caregiver cooked MMT porridge flour 7 days the previous week	MMT #2	76.2% (72.7, 79.6%)	N/A	N/A	N/A	N/A	N/A	N/A	N/A	N/A
Caregiver reported that other household members tasted flours	MMT #1	90.2% (87.6, 92.9%)	N/A	N/A	N/A	N/A	N/A	N/A	N/A	N/A
Caregiver reported sharing flours outside the household	MMT #4	16.2% (12.5, 19.9%)	N/A	N/A	N/A	N/A	N/A	N/A	N/A	N/A
Persistence/sustained behaviors										
Fed cereal/groundnut blended flour porridge	MMT #1	N/A	N/A	N/A	N/A	N/A	N/A	86.5% (83.8, 89.1%)	74.7% (72.0, 77.5%)	<0.01 *
Fed with MMT-produced porridge flour—intervention arm	MMT #2	N/A	N/A	N/A	N/A	N/A	N/A	73.2% (69.7, 76.8%)	N/A	N/A

Note: the number of respondents in the intervention arm varies between 1018 and 1032 because 14 mothers missed monthly gift distribution the month prior to the 12 mo survey. * = *p* value < 0.05. ^†^ General IYCF or specific MMT trial feeding recommendation: MMT #1: Groundnut added to porridge adds protein to help children grow. MMT #2: Porridge flours of cereals with protein are nutritious. IYCF #1: Infants should continue to breastfeed between 6 and 23 mos. IYCF #2: Infants should be fed with the following frequency—breastfed at 12 mos old for 3–4 meals with 1–2 snacks; non-breastfed, 4–5 meals. MMT #3: Infants 12 mos or older should be fed up to 460 mL of porridge/day. IYCF #3: Consume a variety for foods for healthy growth and development. MMT#4: Feed the MMT flours to the index child as much as possible. IYCF—infant and young child feeding; MMT—Mycotoxin Mitigation Trial; N/A—Not applicable based on trial design and/or survey collection.

## Data Availability

Data described in the manuscript, code book, and analytic code will be made publicly and freely available without restriction through the Cornell eCommons Digital Repository. Data will be available upon request from corresponding author.

## Abbreviation

AF	aflatoxin
BCC	behavior change communication
IRB	Institutional Review Board
IYCF	infant and young child feeding
LBW	low birthweight
LNS	lipid-based nutrient supplement
MMT	Mycotoxin Mitigation Trial
NIMR	National Institute for Medical Research
PIP	Program Impact Pathway
SoC	standard of care
USDA	United States Department of Agriculture
